# Supplementary material for: The psychosocial impact of disability and stigma among persons affected by leprosy and lymphatic filariasis in India
Source: PLOS Ment Health. 2026 Jul 13;3(7):e0000645. doi: 10.1371/journal.pmen.0000645 (PMC13362095; doi:10.1371/journal.pmen.0000645)
Supplement: Attachment — Submitted filename: 260213 Response to reviewers comments.docx [file pmen.0000645.s002.docx]

Dear Editors,

We are pleased to submit the revised version of our manuscript. We have carefully addressed all reviewer comments, as detailed in the accompanying table of responses. In addition, we have addressed the editorial comments provided.

Regarding the striking image included in the manuscript, we confirm that it was originally generated by the authors using ChatGPT. No third-party copyrighted material was used in its creation. Therefore, the image is fully owned by the authors and can be published under the Creative Commons Attribution 4.0 International (CC BY 4.0) License.

We sincerely hope that the revisions and clarifications provided satisfactorily address the comments, and we look forward to your consideration of our revised manuscript.

Thank you for your attention and support.

Sincerely,
Robin van Wijk
On behalf of all co-authors

| Reviewer’s comment | Response |
| --- | --- |
| Reviewer 1 | |
| The text is very clear and easy to read. The methodology is sound and the originality of this text is unambiguous. The results obtained seem revealing to me, given the sample size used. In summary, this study highlights the multiple stigmas faced by people living with leprosy. | Thank you for this feedback. We appreciate the confirmation of urgency of the issue. |
| Reviewer 2 | |
| Overall, the manuscript is well-written, and the study appears to be well-conducted. Only minor corrections are required. | Thank you for the feedback. We have made an effort to address the mentioned issues. |
| References are missing in lines 68 and 71. | Thank you for pointing this out, we have inserted the missing references.  We kindly refer to page 4 in the manuscript. |
| The authors have included community participants only from Bokaro district, with no representation from Jaunpur district. | The inclusion of community members was specifically intended to establish context-specific cut-off values for the mental wellbeing measure, rather than to serve as a fully representative control group for comparative analyses across study sites. Since it was conducted in a later phase of the study, community member recruitment was limited to Bokaro district due to logistical constraints and the availability of complete mapping data at the time of this phase of the study. While Bokaro provided a sufficiently heterogeneous community context for deriving cut-off values, we acknowledge that the absence of community members from Jaunpur limits the generalisability of these cut-offs to that district.  This limitation has now been explicitly acknowledged in the discussion section. Lines 401-407  *The inclusion of community members was specifically intended to establish context-specific cut-off values for the WEMWBS, rather than to serve as a fully representative control group for comparative analyses across study sites. Consequently, the sample size and sampling strategy were not designed to support in-depth case–control comparisons. While Bokaro provided a sufficiently heterogeneous community context for deriving cut-off values, we acknowledge that the absence of community members from Jaunpur limits the generalisability of these cut-offs to that district.* |
| In education, the author needs to explain tertiary education, as it is not clear whether it refers to undergraduate (UG), postgraduate (PG), or doctoral (PhD) levels. | Thank you for pointing this out, we have added this below the relevant table. Line 244  ** Tertiary education includes undergraduate, postgraduate, as well as doctoral levels* |
| It would be good if the author discussed the results from Table 4 in the discussion section. | We thank the reviewer for this helpful suggestion. Table 4 presents the results of the univariate analyses between scale outcomes and sociodemographic and disability-related variables. These analyses were conducted primarily to explore crude associations and to inform the selection of variables for inclusion in the multivariate models presented in Table 5.  As such, the univariate results in Table 4 are intended to provide descriptive and exploratory detail for the reader, illustrating mean differences across subgroups, rather than to support independent inferential conclusions. The main interpretative focus of the study is therefore placed on the multivariate analyses, which account for potential confounding and are discussed in detail in the discussion section.  To avoid redundancy and overinterpretation of unadjusted associations, we did not include a separate discussion of the univariate findings. |
| Reviewer 3 | |
| Introduction |  |
| In the introduction section instead of citing reference you put it as ‘ref’ | Thank you for pointing this out, we have inserted the missing references. |
| The references are put after the end of sentence by full stop. | Thank you, we have tracked this throughout the entire paper and made corrections where necessary. |
| The last sentence of the introduction which is the objective do not align with the title of the study | We thank the reviewer for this comment. We agree that the study objective can be more explicitly aligned with the title. While social participation and mental wellbeing were intended as operational components of psychosocial impact, this was not sufficiently clear in the original wording. The objective has therefore been revised to explicitly refer to the psychosocial impact of disability and stigma, in line with the title of the manuscript. Lines 114-117  *The present study aimed to examine the psychosocial impact of disability and stigma on social participation and mental wellbeing among persons with leprosy- and LF-related disabilities in Jaunpur in Uttar Pradesh and Bokaro in Jharkhand in India*. |
| Methodology |  |
| Why you select this districts for the study : Jaunpur, Uttar Pradesh and Bokaro in Jharkhand | We thank the reviewer for this question. Jaunpur (Uttar Pradesh) and Bokaro (Jharkhand) were selected based on their high endemicity for both leprosy and LF, ensuring the feasibility of recruiting sufficient numbers of persons affected by both conditions. In 2019, the NVBDCP reported 21,615 persons with LF-related symptoms in Bokaro and 10,823 in Jaunpur, indicating a substantial burden in both districts. In addition, the proximity of Bokaro to Ranchi, where the Central Institute of Psychiatry (CIP) is located, allowed access to specialised mental health expertise and support for the broader project within which this study was embedded. These considerations together informed the selection of the two study districts.  This rationale for choosing these study areas was added to the methods section. Lines 132-134  *Jaunpur district in Uttar Pradesh and Bokaro district in Jharkhand were purposively selected due to their high endemicity for both leprosy and lymphatic filariasis, which ensured the relevance of the area, and feasibility of recruiting sufficient numbers of persons affected by both conditions.* |
| The data collection was done between 18 October 2021 and December 2021. Why is the delay in submission for publication for almost 4 years. | We thank the reviewer for raising this point and acknowledge the delay between data collection and submission. This study formed part of a larger research programme, and priority was initially given to analysing and publishing the primary outcomes related to the intervention, which are referenced in the discussion section of the current manuscript. The preparation of the present paper therefore followed the completion of those analyses. In addition, the timeline was affected by unforeseen personal circumstances of the first author, which temporarily slowed manuscript development. We regret the delay and appreciate the opportunity to publish these findings, which remain relevant given the persistent burden of leprosy- and LF-related disability and stigma in the study settings. |
| Lines 124-126. Why random sampling was taken among patients ,where as purposive sampling in apparently healthy community members | We appreciate this question and the opportunity to clarify the rationale. Random sampling was applied among persons affected by leprosy and LF to minimise selection bias and ensure representativeness within the defined sampling frame of affected persons with disabilities. In contrast, purposive sampling was used for community members because their role in the study was methodological rather than comparative. Community members were selected from blocks with the highest concentration of affected persons to ensure exposure to similar socio-economic and environmental conditions, while deliberately excluding individuals with close personal ties to affected persons (see response below). This approach allowed us to obtain a contextually relevant reference group for mental wellbeing scoring rather than a statistically matched control group. Such mixed sampling strategies are commonly used in public health studies when different participant groups serve distinct analytical purposes. |
| Line 132-133. What is the justification for excluding the family members, relatives and direct neighbours ? | Family members, relatives, and direct neighbours were intentionally excluded to avoid stigma by association, a well-documented phenomenon whereby people closely connected to persons affected by leprosy or LF experience secondary stigma, social exclusion, and psychological distress themselves. In addition, close family members and neighbours often take on informal caregiving roles, which may introduce mental health stressors related to caregiving burden rather than reflecting the mental wellbeing of the general community. Including such participants would therefore risk misclassifying community mental wellbeing and confounding the interpretation of results. By excluding these groups, we aimed to ensure that community member data reflected mental wellbeing unaffected by direct caregiving responsibilities or associative stigma, thereby strengthening the validity of the reference values derived. |
| Line 140. The assumption of 50% having poor mental wellbeing looks exaggerated, it is good to check litrature | This approach is recommended when prior prevalence estimates are unavailable or uncertain. (1) We thank the reviewer for this comment. The assumption of 50% prevalence for poor mental wellbeing was not based on prior literature but was intentionally chosen as a conservative estimate to ensure the largest required sample size, thus minimising sampling errors. In sample size calculations for prevalence studies, assuming a prevalence of 50% maximizes the product p(1−p), resulting in the largest possible sample size for a given precision. We have now added this motivation to the manuscript. Lines 160-162  *The assumed prevalence of poor mental wellbeing was set at 50% to provide a conservative estimate, as this maximizes the required sample size when prior prevalence data are unavailable.*  1. Naing, L., Winn, T., & Rusli, B. N. (2006). Practical issues in calculating the sample size for prevalence studies. Archives of Orofacial Sciences, 1, 9–14. |
| Why is the proportion of controls very low?  It is written as total of 201 persons with leprosy-related disabilities, 240 with LF-related disabilities, and 98 non-affected community members were included It should have been at least 1:1, what is the justification of not including reasonable number of controls | We thank the reviewer for raising this important point. The community member sample was not intended to function as a traditional control group for case–control comparisons. Instead, it was included to (1) describe mental wellbeing in a non-affected community context and (2) derive context-specific cut-off values for the mental wellbeing instrument. A 1:1 ratio between cases and controls is not a methodological requirement when the objective is not hypothesis testing between groups. Epidemiological and biostatistical literature indicates that increasing the number of controls beyond what is needed for the intended analysis yields diminishing returns in precision, particularly when controls are used for calibration or descriptive purposes rather than effect estimation. (2) Given the specific analytical purpose of the community member data, a smaller sample was sufficient. However, we acknowledge that the unequal group sizes limit the use of community members for robust comparative analyses, and this has now been clearly stated as a limitation in the manuscript. Lines 401-407  *The inclusion of community members was conducted in a later phase of the study and was specifically intended to establish context-specific cut-off values for the WEMWBS, rather than to serve as a fully representative control group for comparative analyses across study sites. Consequently, the sample size and sampling strategy were not designed to support in-depth case–control comparisons. While Bokaro provided a sufficiently heterogeneous community context for deriving cut-off values, we acknowledge that the absence of community members from Jaunpur limits the generalisability of these cut-offs to that district.*  2. Rothman, K.J., Greenland, S., & Lash, T.L. (2008). Modern Epidemiology (3rd ed.). Lippincott Williams & Wilkins. |
| Why R2 as a measure of statistics | We thank the reviewer for this question. R² is reported to indicate the proportion of variance in the outcome variable explained by the predictors in the multivariate model. This provides readers with a sense of the explanatory power of the model alongside the significance of individual predictors. We have added this explanation to the analysis paragraph in the method’s section of the paper. Lines 211-212  *When reporting, we included R² to provide an overall measure of the model’s explanatory power, complementing the interpretation of individual regression coefficients*. |
| Results |  |
| Line 211, Just out of curiosity why is 42% of cases have grade II disability? It is far above the global average. | We thank the reviewer for this observation. It is important to note that the inclusion criteria for this study required participants to have at least one form of disability. As a result, persons affected by leprosy without disability were not included, which naturally increases the proportion of participants with grade 2 disabilities compared to global averages that include all persons affected, regardless of disability status. Additionally, the study was conducted in endemic areas where access to timely care may be limited, and the prevalence of more severe disabilities is consequently higher. The observed 42% of participants with grade 2 disability is therefore consistent with the study’s sampling framework and the local context. |
| Table 2. Nearly 25% of healthy controls have mild depression and 14% has moderate depression. How do you explain this? The same is true for 35.5% of mild participation restriction. | We thank the reviewer for this observation. Depression and participation restrictions in community members are influenced by a range of factors beyond leprosy or LF. In low-resource settings such as the study districts, social and economic challenges, including poverty, limited employment opportunities, and household stressors, can contribute to depressive symptoms. Similarly, limitations in social participation may arise from general social, work, school, or family-related barriers, independent of disease status.  Acknowledging these findings highlights that not all differences in mental wellbeing or participation outcomes can be attributed solely to leprosy or LF, and underscores the importance of considering broader contextual and socio-economic factors when interpreting the results. We have added a sentence to the discussion section to emphasise this finding. Lines 329-331  *Some depression and participation restrictions were observed among non-affected community members, reflecting the influence of broader socio-economic and contextual factors, rather than being attributable solely to leprosy or LF.* |
| Lines 271-273. Why is there Negative associations between stigma and depression, and also stigma and participation restriction in both disease groups were observed. Scientifically as stigma increases both depression and participation increases | We thank the reviewer for pointing this out. We apologise for any confusion caused by the phrasing in the manuscript. What we intended to convey is that higher stigma scores are associated with higher depression scores and higher participation restriction scores, that is, stigma has a detrimental (negative) influence on mental wellbeing and participation. Lines 303-305  *Higher stigma scores were associated with higher depression scores and greater participation restrictions in both disease groups (Table 5), indicating the detrimental impact of stigma on psychosocial outcomes.* |
| Conclusion |  |
| Line 376-378, which is the last sentence of the conclusion “ Further research into the connections between education, disability, and mental health is essential to deepen understanding of these complex intersections and to guide the development of more effective, inclusive interventions.” This by itself is not enough to address the outcomes identified in the study. Having all sorts of impaired psychosocial and mental wellbeing outcomes, I suggest future studies should focus on assessing the impact of psychosocial interventions in alleviating stigma and improving quality of life and mental wellbeing. | We thank the reviewer for this suggestion. We agree that the original wording of the final sentence could be more directly aligned with the study’s main outcomes. The sentence has been revised to emphasise the need for future research on psychosocial interventions, including stigma reduction and peer-support programmes, to improve mental wellbeing, quality of life, and social participation among persons affected by leprosy and lymphatic filariasis. Lines 424-427  *Further research should focus on assessing the impact of psychosocial interventions, including stigma reduction and peer-support programmes, on improving mental wellbeing, quality of life, and social participation among persons affected by leprosy and lymphatic filariasis.* |
