# Supplementary material for: The psychosocial impact of disability and stigma among persons affected by leprosy and lymphatic filariasis in India
Source: PLOS Ment Health. 2026 Jul 13;3(7):e0000645. doi: 10.1371/journal.pmen.0000645 (PMC13362095; doi:10.1371/journal.pmen.0000645)
Supplement: Attachment — Submitted filename: 260521 Response to reviewers comments.docx [file pmen.0000645.s003.docx]

Dear Editors,

We are pleased to submit the revised version of our manuscript. We have carefully addressed all reviewer comments, as detailed in the accompanying table of responses.

We sincerely hope that the revisions and clarifications provided satisfactorily address the comments, and we look forward to your consideration of our revised manuscript.

Thank you for your attention and support.

Sincerely,
Robin van Wijk
On behalf of all co-authors

| Reviewer’s comment | Response |
| --- | --- |
| Reviewer 4 |  |
| The sampling combines purposive (district selection, community controls) and random approaches (cases). Community controls were recruited only in Bokaro, while cases came from both Bokaro and Jaunpur. This may introduce a selection bias and limit the comparison. There needs to be a line of justification. | The following is included in the discussion section, addressing the reviewer’s concerns: Line 424 - 433  *The inclusion of community members was specifically intended to establish context-specific cut-off values for the WEMWBS, rather than to serve as a fully representative control group for comparative analyses across study sites. Consequently, the sample size and sampling strategy were not designed to support in-depth case–control comparisons. While Bokaro provided a sufficiently heterogeneous community context for deriving cut-off values, we acknowledge that the absence of community members from Jaunpur limits the generalisability of these cut-offs to that district*. |
| A conceptual framework stating the relationships between stigma, depression, participation, and mental wellbeing will improve the interpretations. | Thank you for pointing that out. We have now made reference to a framework of How are NTDs, stigma and mental health connected? By Eaton, 2017. Lines 113-116  *Eaton describes a cyclical relationship between stigma, mental distress (including depression and reduced mental wellbeing), and social participation, whereby stigma contributes to psychological burden and restricted participation, which in turn can reinforce stigma and adverse health outcomes* |
| Mention who administered the tools and what was the language used. | We have added this in line 217-218  *Data was collected by trained research assistants, fluent in both Hindi and other local languages and dialects.* |
| Explain the finding higher education associated with poorer mental wellbeing (leprosy group) - suggestions- increased awareness and/or unmet expectations. Explain leprosy shows higher stigma; LF shows different stigma domains. | Thank you for this insightful comment. We have carefully considered this finding and explored potential explanations, including the suggested factors such as increased awareness and unmet expectations. However, we did not identify supporting evidence in the literature, nor did our engagement with the study population provide clear indications to substantiate these interpretations. We have therefore chosen not to speculate and instead recommend further research to better understand this association. |
| recommendations could be more specific and actionable. | Thank you for this helpful suggestion. We have revised the Conclusion section to provide more specific and actionable recommendations, including the integration of psychosocial support, stigma reduction, peer counselling, community awareness raising, family engagement, and socio-economic support within NTD programmes.  Line 450-453  *Comprehensive and person-centred approaches that combine psychosocial support, stigma reduction, peer counselling, community awareness raising, family engagement, and socio-economic support may help improve wellbeing and social inclusion among persons affected by NTDs.* |
|  |  |
| Reviewer 5 |  |
| Are there specific impacts, stigmas, or previous reports regarding disability in Bokaro and Jaunpur districts that justify selecting these locations? If so, please include them in the introduction or the methods section. | Thank you for this important suggestion. While no published studies were identified that specifically address stigma or disability in Bokaro and Jaunpur districts, the selection of these sites was informed by their high prevalence of leprosy and LF-related disabilities. In addition, evidence from Deoghar district in the same state (Jharkhand) demonstrates significant knowledge gaps, persistent misconceptions, and stigma related to leprosy, which affect health-seeking behaviours (Sree et al., 2025). We have now included this contextual information in the Methods section to better justify the selection of the study areas.  Lines 153-156  *While no prior studies have specifically examined stigma and disability in Bokaro and Jaunpur districts, evidence from Deoghar district in Jharkhand State highlights substantial knowledge gaps, persistent misconceptions, and stigma related to leprosy, which negatively influence health-seeking behaviours and access to care.* |
| • Why was the additional study for community members performed only in the Bokaro district? | The following is included in the discussion section, addressing the reviewer’s question: Line 424 -433  *The inclusion of community members was specifically intended to establish context-specific cut-off values for the WEMWBS, rather than to serve as a fully representative control group for comparative analyses across study sites. Consequently, the sample size and sampling strategy were not designed to support in-depth case–control comparisons. While Bokaro provided a sufficiently heterogeneous community context for deriving cut-off values, we acknowledge that the absence of community members from Jaunpur limits the generalisability of these cut-offs to that district*. |
| • Given the total of 57 items across the four measuring instruments, what was the estimated data collection time per participant? Is it possible to mention in the manuscript? | Thank you for this helpful suggestion. The exact duration of data collection per participant was not systematically recorded, and we are unfortunately unable to retrieve this information retrospectively. We acknowledge that including this detail would improve clarity and will consider this in future studies. |
|  |  |
| Reviewer 6 |  |
| Line 64: It would be better to add 'psychological' to mean medical and social support. This is because your title mainly focuses on psychosocial issues. | Thank you for pointing this out. We have added the word to the sentence.  Line 62 |
| Line 68: According to the reference, there are 22 NTDs rather than 21. It would be better to correct this data. | Thank you for pointing this out. We note that the number of NTDs was updated from 21 to 22 after the initial submission of the manuscript, and we have now revised the text accordingly.  Line 62  *A more holistic approach, bringing together medical, psychological and social support, can help people with neglected tropical diseases live healthier and more fulfilling lives.* |
| Line 83: It is very important to describe that parasite. Therefore, it is valuable to add Wuchereria bancrofti, Brugia malayi, and Brugia timori. | Thank you, we have added the information to the relevant sentence. Line 81-82 *LF, also known as elephantiasis, is caused by filarial parasites such as Wuchereria bancrofti, Brugia malayi, and Brugia timori, which are transmitted by mosquitoes.* |
| Line 85: It would be better to say 'the parasite' instead of 'the diseases.' | Thank you, this makes sense. We have adjusted accordingly.  Line 83 |
| Line 86: Make it clear that LF also affects the arm and breast, depending on the affected site. | Thank you for pointing this out. We have added ‘breasts’ to the sentence for clarity. While the term ‘limbs’ includes both upper and lower limbs, we agree that specifying additional affected sites improves the precision of the description.  Line 85 |
| Lines 87 and 88 need a reference. | Thank you for pointing this out. We have now included the relevant reference to support these statements.  Line 88-89 |
| Line 132: Study design. This includes the study setting and period, so include these in the subtitle. | Ok Line 133 |
| Line 134: Does your study design indicate an analysis of psychosocial outcomes and the influence of disability and stigma? It would be better to consider the difference between correlation and causation here. | Thank you for this important observation. We agree that the study design does not allow conclusions regarding causality. We have therefore revised the wording in the manuscript to refer to associations rather than influence or impact. We have also further scanned the document and adjusted wording directly indicating causation.  Line 134-136  *The design allowed analysis of psychosocial outcomes and their association with disability and stigma among persons affected by leprosy and LF in the Bokaro (Jharkhand) and Jaunpur (Uttar Pradesh) districts in India.* |
| Line 137: You purposively selected two areas due to their high endemicity. Do you think the results can be generalized to low-endemic areas as well? | Thank you for this important observation. As the study was conducted in high-endemic districts purposively selected for their disease burden, the findings may not be fully generalisable to low-endemic settings, where experiences of stigma and psychosocial outcomes may differ. We have acknowledged this in the Discussion section as a limitation of the study. Line 416-418 *In addition, the study was conducted in purposively selected high-endemic districts, and the findings may therefore not be fully generalisable to low-endemic settings, where disease visibility, community perceptions, and experiences of stigma may differ.* |
| It would be better to change the location of lines 140–143 to the data collection section/subtitle, unless you have a concrete reason for including them here. | Thank you for this suggestion. We have moved this information to the data collection section for improved clarity and structure.  Line .. |
| Line 149: What type of random sampling did you use for the selection? Additionally, how many people with disabilities were found in the two districts, and how was the collected sample allocated to each site? This needs further clarification. | Thank you for this comment. We have clarified the sampling procedure in the Methods section by specifying that, when the number of eligible participants exceeded the required sample size, respondents were selected through a draw of lots. We have also clarified the allocation of the sample across the study sites in the manuscript.  Line 160-163  *Within each village, all persons with leprosy- or LF-related disabilities were invited to participate in the study. In instances where the number of eligible persons exceeded the required sample size, respondents were selected through a draw of lots by randomly selecting numbered folded papers.* |
| Line 154: What impact does excluding people with serious mental health problems have on the validity and results? | Thank you for this important observation. Persons with severe mental health problems who were unable to respond to the interview questions were excluded to ensure informed participation and the reliability of responses. We acknowledge that this exclusion may have led to an underestimation of psychosocial burden within the study population and have noted this as a limitation in the Discussion section.  Line 441-443  *Finally, although persons with severe mental health problems who were unable to participate in interviews were excluded, this was not expected to substantially affect the findings, as only a limited number of such cases were encountered during data collection*. |
| Line 156: Only community members were selected by gender. Do you think age and educational level are irrelevant factors in the validity of the control? Why not consider these factors as well? | Thank you for this important observation. We agree that age and educational level may also influence psychosocial outcomes and could have strengthened comparability between groups. However, in this study, community members were selected to achieve gender comparability with affected persons, and additional matching factors were not included. We have acknowledged this as a limitation in the Discussion section.  Line 427-430  *Community members were selected to achieve gender comparability with affected persons; however, other potentially relevant factors, such as age and educational level, were not considered and may have influenced comparisons between groups.* |
| Line 159: Why were family members, relatives, and direct neighbors excluded? | Thank you for this important question. Family members, relatives, and direct neighbours were excluded from the community sample because their close association with persons affected by leprosy or LF could influence their perceptions, psychosocial wellbeing, and experiences of stigma through associative or courtesy stigma. Excluding these groups was intended to reduce potential bias in the community comparison group.  Line 174-176  *Family members, relatives or direct neighbours were excluded from the community sample to minimise the potential influence of associative stigma related to leprosy or LF.* |
| Line 164: Why were community members selected only in the Bokaro district? How does this align with the context-specific cut-off values for this measurement presented on line 142? | Thank you for this observation. We have clarified this issue further in the Discussion section, including the rationale for selecting community members only in Bokaro and the implications for the context-specific WEMWBS cut-off values and generalisability of the findings. |
| Line 200: Delete one of the 35 at the end of the sentence. | Thank you for pointing this out. We were unable to identify this issue in the current version of the manuscript and suspect it may relate to tracked changes or a previous draft version. |
| In Table 1: Please delete the 'grade 0 disability' row value. To be consistent, change 'n/a' to 'N/A'. | Thank you for this observation. We have revised Table 1 accordingly by removing the ‘grade 0 disability’ row value and changing ‘n/a’ to ‘N/A’ for consistency. |
| Line 252. In what ways do the psychometric properties and score distributions of the Participation Scale (PSSS) differ from those of the PHQ-9? How does this influence the choice between mean and median as a measure of central tendency? | Thank you for this comment. The PSSS scores were not normally distributed and were therefore summarised using the median and interquartile range, whereas the PHQ-9 and other scale scores were approximately normally distributed and could therefore be summarised using the mean and standard deviation. As this reflects standard statistical practice, we did not consider further elaboration necessary in the manuscript. |
| Line 255: Why are you using the median for the PSSS? | The median and interquartile range were used for the PSSS because the score distribution was non-normal. |
| In Table 2: Why is it important to report the mean with CI and the median with IQR in the same table? | Mean with confidence interval and median with interquartile range were reported according to the distribution characteristics of the variables. We regard this as standard statistical practice and therefore did not provide further elaboration in the manuscript. |
| Lines 259–260: Why is the median used for SSS? | The PSSS scores were not normally distributed and were therefore summarised using the median and interquartile range. |
| Line 264: It is better to report by grade number than to report lower lymphoedema grades. | Thank you for this suggestion. However, we retained the current wording because it reflects the outcome of the statistical analysis, which identified a significant difference between persons with lymphoedema grade 3 and those with lower lymphoedema grades. |
| In Table 3, why is the mean per question reported for experienced stigma, disclosure concern, internalized stigma, and anticipated stigma, rather than the mean of all questions and the median of each subscale? In addition, when reporting these in the table, it is preferable to describe the lower and upper ranges of the mean subscale scores in the Methods section. | Thank you for this comment. Mean subscale scores were reported to maintain comparability across the different domains within the same stigma scale and to facilitate interpretation alongside the overall scale score. While some subscales contained relatively few items, the overall scale scores were approximately normally distributed, and we therefore considered reporting mean subscale scores appropriate. We did not include the lower and upper ranges of the subscale scores in the Methods section to avoid unnecessary complexity and maintain readability. |
| Line 277: It says, 'Associations between the psychosocial outcomes and stigma are visualized in Figure 1,' but, as described above, the figure is not included in the document. | Thank you for this observation. Following the journal’s submission guidelines, figures were submitted separately from the main manuscript file. Figure 1 was therefore included as a separate upload accompanying the submission. |
| From line 278: I understand that there is no association between stigma and mental well-being and that persons affected by leprosy are indirectly part of the description. If so, it would be better to describe this in the final part of the paragraph alongside its P-value. | Thank you for this observation. The paragraph refers specifically to persons affected by leprosy, as indicated by the subsection heading and first sentence of the paragraph. However, we agree that the absence of a significant association between stigma and mental wellbeing could be stated more explicitly, and we have clarified this in the text by including the corresponding p-value.  Lines 301-302  *No significant association was found between stigma and mental wellbeing (p=0.855).* |
| However, in lines 290 and 291. I have seen the reputation of ideas for people affected by leprosy, stigma, and depression under the 'Persons Affected by Lymphatic Filariasis' sub-section. So, delete this part. | Thank you for this observation. We intended to indicate that similar associations were observed among persons affected by LF as among persons affected by leprosy. To improve clarity, we have revised the wording in this section.  Lines 308-310  *Associations between stigma and depression (p<0.001), as well as stigma and participation restriction (p<0.001), were also observed among persons affected by LF, similar to the findings for persons affected by leprosy.* |
| Line 293 or Table 4. It is good to present it in landscape format, and for the mean and median, present one row for each variable and one line for the confidence interval (CI) and interquartile range (IQR) accordingly. To avoid writing sociodemographic variables twice for persons affected by leprosy and LF, it is better to write them in front of the first column: sex, age, marital status, and so on. This will help readers to grasp the information easily, as they need a clear table presentation. | Thank you for these suggestions. We acknowledge that Table 4 contains a substantial amount of information. However, the current structure was intentionally chosen to present the separate multivariate models for each disease group and psychosocial outcome as clearly and accurately as possible. Sociodemographic variables are reported only when statistically significant within a specific model, which results in some repetition across models. We believe that the current format provides the most transparent representation of the analyses while maintaining interpretability of the multivariate results. Nevertheless, we carefully reviewed the table layout to improve readability where possible. |
| Again, for the Table 4 result, you have not described what type of statistical analysis you used. | Thank you for these suggestions. Mean with confidence interval and median with interquartile range were presented according to the distribution characteristics of the variables, following standard statistical practice. To maintain readability and avoid unnecessary repetition, we did not report both summary measures for each outcome. In addition, the placement of the sociodemographic variables within the table was a deliberate formatting choice intended to balance clarity and table density. After careful consideration, we believe the current structure provides the clearest presentation of the data. |
| Line 313: Table 5. Why is a multilevel model being run for a combined sample? How do you categorize visible and non-visible disabilities here? Also, why do you use the symbol β instead of B for beta? | Thank you for these observations. Table 5 presents separate multivariate regression models for persons affected by leprosy and persons affected by LF; no combined sample or multilevel analysis was used. Visible and non-visible disabilities were categorised according to the established disability classifications. In addition, the symbol β was used to denote the regression coefficient, following standard statistical notation. |
| Line 358: Koschorke et al. is reference #42, not #41. Reference #41 is by Somar, Waltz, and van Brakel. | Thank you for pointing this out. We reviewed and corrected the reference list and citation links, including the citation numbering in this section. |
| Lines 421 – 428. It is vital to address mental well-being, depression, restricted participation, and stigma by integrating the various intervention methods you listed. However, these are not the only interventions. As previously mentioned, psychosocial problems are related to disability and stigma. Therefore, raising awareness and implementing religious, psychosocial, economic, holistic, and family-based interventions can significantly alleviate such problems, as evidenced by research conducted in various areas. As a recommendation, it would be better to make the approach more comprehensive. | Thank you for this important observation. We agree that addressing psychosocial problems among persons affected by leprosy and LF requires a comprehensive and holistic approach that extends beyond mental health and stigma reduction alone. We have therefore revised the Conclusion section to incorporate broader community-based, psychosocial, family-oriented, and socio-economic approaches, alongside peer support and awareness-raising interventions, to better reflect the multifaceted nature of psychosocial wellbeing and social inclusion.  Lines 450-453  *Comprehensive and person-centred approaches that combine psychosocial support, stigma reduction, peer counselling, community awareness raising, family engagement, and socio-economic support may help improve wellbeing and social inclusion among persons affected by NTDs.*  And line 459  *Further research should focus on assessing the impact of psychosocial interventions, including stigma reduction and peer-support programmes, on improving mental wellbeing, quality of life, and social participation among persons affected by leprosy and lymphatic filariasis across different social, cultural, and community contexts.* |
