# Supplementary material for: The psychosocial impact of disability and stigma among persons affected by leprosy and lymphatic filariasis in India
Source: PLOS Ment Health. 2026 Jul 13;3(7):e0000645. doi: 10.1371/journal.pmen.0000645 (PMC13362095; doi:10.1371/journal.pmen.0000645)
Supplement: Peer review history

# Author comment: Author response to Decision Letter 0

**DOI:** 10.1371/journal.pmen.0000645.r001

**Transfer Alert**

This paper was transferred from another journal. As a result, its full editorial history (including decision letters, peer reviews and author responses) may not be present.

23 Oct 2025

# Peer review: Decision Letter 0

**DOI:** 10.1371/journal.pmen.0000645.r002

**Authors:** Emma Campbell

8 Jan 2026

PMEN-D-25-00458

The psychosocial impact of disability and stigma among persons affected by leprosy and lymphatic filariasis in India

PLOS Mental Health

Dear Dr. van Wijk,

Thank you for submitting your manuscript to PLOS Mental Health. After careful consideration, we feel that it has merit but does not fully meet PLOS Mental Health’s publication criteria as it currently stands. Therefore, we invite you to submit a revised version of the manuscript that addresses the points raised during the review process.

The manuscript has been assessed by three reviewers and their comments can be found below and in the attached document. The reviewers have requested some further detail in the methodology and additional clarification and discussion of some of the results. Please review their comments and make the appropriate revisions to your manuscript.

Please submit your revised manuscript by Feb 21 2026 11:59PM. If you will need more time than this to complete your revisions, please reply to this message or contact the journal office at mentalhealth@plos.org. When you're ready to submit your revision, log on to https://www.editorialmanager.com/pmen/ and select the 'Submissions Needing Revision' folder to locate your manuscript file.

Please include the following items when submitting your revised manuscript:

- A letter that responds to each point raised by the editor and reviewer(s). You should upload this letter as a separate file labeled 'Response to Reviewers'.
- A marked-up copy of your manuscript that highlights changes made to the original version. You should upload this as a separate file labeled 'Revised Manuscript with Track Changes'.
- An unmarked version of your revised paper without tracked changes. You should upload this as a separate file labeled 'Manuscript'.

Guidelines for resubmitting your figure files are available below the reviewer comments at the end of this letter.

We look forward to receiving your revised manuscript.

Kind regards,

Emma Campbell, Ph.D

Staff Editor

PLOS Mental Health

Journal Requirements:

1. Please include a complete copy of PLOS’ questionnaire on inclusivity in global research in your revised manuscript. Our policy for research in this area aims to improve transparency in the reporting of research performed outside of researchers’ own country or community. The policy applies to researchers who have travelled to a different country to conduct research, research with Indigenous populations or their lands, and research on cultural artefacts. The questionnaire can also be requested at the journal’s discretion for any other submissions, even if these conditions are not met.  Please find more information on the policy and a link to download a blank copy of the questionnaire here: https://journals.plos.org/mentalhealth/s/best-practices-in-research-reporting. Please upload a completed version of your questionnaire as Supporting Information when you resubmit your manuscript.

2. Please amend your detailed Financial Disclosure statement. This is published with the article. It must therefore be completed in full sentences and contain the exact wording you wish to be published.

i. Please clarify all sources of financial support for your study. List the grants, grant numbers, and organizations that funded your study, including funding received from your institution. Please note that suppliers of material support, including research materials, should be recognized in the Acknowledgements section rather than in the Financial Disclosure.

ii. State the initials, alongside each funding source, of each author to receive each grant. For example: "This work was supported by the National Institutes of Health (####### to AM; ###### to CJ) and the National Science Foundation (###### to AM)."

iii. State what role the funders took in the study. If the funders had no role in your study, please state: “The funders had no role in study design, data collection and analysis, decision to publish, or preparation of the manuscript.”

iv. If any authors received a salary from any of your funders, please state which authors and which funders.

3. Please ensure that your Ethics Statement is available in its entirety at the beginning of your Methods section, under a subheading 'Ethics Statement'.

4. Please upload separate figure files in .tif or .eps format. Also, remove the figures from your manuscript file but keep the legends.

For more information about figure files please see our guidelines:

https://journals.plos.org/mentalhealth/s/figures

https://journals.plos.org/mentalhealth/s/figures#loc-file-requirements

5. Some material included in your submission may be copyrighted. According to PLOS’s copyright policy, authors who use figures or other material (e.g., graphics, clipart, maps) from another author or copyright holder must demonstrate or obtain permission to publish this material under the Creative Commons Attribution 4.0 International (CC BY 4.0) License used by PLOS journals. Please closely review the details of PLOS’s copyright requirements here: PLOS Licenses and Copyright. If you need to request permissions from a copyright holder, you may use PLOS's Copyright Content Permission form.

Please respond directly to this email or email the journal office and provide any known details concerning your material's license terms and permissions required for reuse, even if you have not yet obtained copyright permissions or are unsure of your material's copyright compatibility.

Potential Copyright Issues:

a. Striking image: Please confirm whether you drew the images / clip-art within the figure panels by hand. If you did not draw the images, please provide (a) a link to the source of the images or icons and their license / terms of use; or (b) written permission from the copyright holder to publish the images or icons under our CC-BY 4.0 license. Alternatively, you may replace the images with open source alternatives. See these open source resources you may use to replace images / clip-art:

- https://commons.wikimedia.org

- https://openclipart.org/

If the reviewer comments include a recommendation to cite specific previously published works, please review and evaluate these publications to determine whether they are relevant and should be cited. There is no requirement to cite these works unless the editor has indicated otherwise.

Additional Editor Comments (if provided):

[Note: HTML markup is below. Please do not edit.]

Reviewers' comments:

Reviewer's Responses to Questions

**Comments to the Author**

1. Does this manuscript meet PLOS Mental Health’s publication criteria? Is the manuscript technically sound, and do the data support the conclusions? The manuscript must describe methodologically and ethically rigorous research with conclusions that are appropriately drawn based on the data presented.

Reviewer #1: Yes

Reviewer #2: Yes

Reviewer #3: Partly

**********

2. Has the statistical analysis been performed appropriately and rigorously?

Reviewer #1: Yes

Reviewer #2: Yes

Reviewer #3: Yes

**********

3. Have the authors made all data underlying the findings in their manuscript fully available (please refer to the Data Availability Statement at the start of the manuscript PDF file)?

The PLOS Data policy requires authors to make all data underlying the findings described in their manuscript fully available without restriction, with rare exception. The data should be provided as part of the manuscript or its supporting information, or deposited to a public repository. For example, in addition to summary statistics, the data points behind means, medians and variance measures should be available. If there are restrictions on publicly sharing data—e.g. participant privacy or use of data from a third party—those must be specified.

Reviewer #1: Yes

Reviewer #2: Yes

Reviewer #3: Yes

**********

4. Is the manuscript presented in an intelligible fashion and written in standard English?

PLOS Mental Health does not copyedit accepted manuscripts, so the language in submitted articles must be clear, correct, and unambiguous. Any typographical or grammatical errors should be corrected at revision, so please note any specific errors here.

Reviewer #1: Yes

Reviewer #2: Yes

Reviewer #3: Yes

**********

5. Review Comments to the Author

Please use the space provided to explain your answers to the questions above. You may also include additional comments for the author, including concerns about dual publication, research ethics, or publication ethics. (Please upload your review as an attachment if it exceeds 20,000 characters)

Reviewer #1: The text is very clear and easy to read. The methodology is sound and the originality of this text is unambiguous. The results obtained seem revealing to me, given the sample size used. In summary, this study highlights the multiple stigmas faced by people living with leprosy.

Reviewer #2: Overall, the manuscript is well-written, and the study appears to be well-conducted. Only minor corrections are required.

References are missing in lines 68 and 71.

The authors have included community participants only from Bokaro district, with no representation from Jaunpur district.

In education, the author needs to explain tertiary education, as it is not clear whether it refers to undergraduate (UG), postgraduate (PG), or doctoral (PhD) levels.

It would be good if the author discussed the results from Table 4 in the discussion section.

Reviewer #3: Comments

Title The psychosocial 1 impact of disability and stigma among persons

2 affected by leprosy and lymphatic filariasis in India

Introduction

1. In the introduction section instead of citing reference you put it as ‘ref’

Leprosy and lymphatic filariasis (LF) are both among the group of NTDs (1). Leprosy is caused by the 71 mycobacterium leprae and has a global incidence of more than 200,000 per year ref.

2. The references are put after the end of sentence by full stop.

motor function in mainly the hands and feet, and blindness. (4,5)

the same is true in other references; 6,7, 8, etc

objective

3. The last sentence of the introduction which is the objective do not align with the title of the study (Line 16 to 108)

The present study aimed to examine the extent to which stigma affects social participation and mental wellbeing among persons with leprosy- and LF-related disabilities in Jaunpur

in Uttar Pradesh and Bokaro in Jharkhand in India.

Methods

4. Why you select this districts for the study : Jaunpur, Uttar Pradesh and Bokaro in Jharkhand ?

Line 116. The data collection was done between 18 October 2021 and December 2021. Why is the delay in submission for publication for almost 4 years.

5. Lines 124-126. Why random sampling was taken among patients ,where as purposive sampling in apparently healthy community members

6. Line 132-133. What is the justification for excluding the family members, relatives and direct neighbours ?

7. Line 140. The assumption of 50% having poor mental wellbeing looks exaggerated, it is good to check litrature

8. Why is the proportion of controls very low?

It is written as total of 201 persons with leprosy-related disabilities, 240 with LF-related disabilities,

and 98 non-affected community members were included

It should have been at least 1:1, what is the justification of not including reasonable number of controls

9. Why R2 as a measure of statistics

Results

10. Line 211, Just out of curiosity why is 42% of cases have grade II disability? It is far above the global average.

11. Table 2. Nearly 25% of healthy controls have mild depression and 14% has moderate depression. How do you explain this?

The same is true for 35.5% of mild participation restriction.

12. Lines 271-273. Why is there Negative associations between stigma and depression, and also stigma and participation restriction in both disease groups were observed.

Scientifically as stigma increases both depression and participation increases

Discussion

It is well outlined and well written

Conclusion

13. Line 376-378, which is the last sentence of the conclusion “ Further research into the connections between education, disability, and mental health is essential to deepen understanding of these complex intersections and to guide the development of more effective, inclusive interventions.”

This by itself is not enough to address the outcomes identified in the study. Having all sorts of impaired psychosocial and mental wellbeing outcomes, I suggest future studies should focus on assessing the impact of psychosocial interventions in alleviating stigma and improving quality of life and mental wellbeing.

**********

6. PLOS authors have the option to publish the peer review history of their article (what does this mean?). If published, this will include your full peer review and any attached files.

**Do you want your identity to be public for this peer review?**  If you choose “no”, your identity will remain anonymous but your review may still be made public.

For information about this choice, including consent withdrawal, please see our Privacy Policy.

Reviewer #1: **Yes:**  Ibrahim MALAM MAMANE SANI

Reviewer #2: No

Reviewer #3: No

**********

[NOTE: If reviewer comments were submitted as an attachment file, they will be attached to this email and accessible via the submission site. Please log into your account, locate the manuscript record, and check for the action link "View Attachments". If this link does not appear, there are no attachment files.]

-->Figure Resubmissions:

While revising your submission, we strongly recommend that you use PLOS’s NAAS tool (https://ngplosjournals.pagemajik.ai/artanalysis) to test your figure files. NAAS can convert your figure files to the TIFF file type and meet basic requirements (such as print size, resolution), or provide you with a report on issues that do not meet our requirements and that NAAS cannot fix.

After uploading your figures to PLOS’s NAAS tool - https://ngplosjournals.pagemajik.ai/artanalysis, NAAS will process the files provided and display the results in the "Uploaded Files" section of the page as the processing is complete. If the uploaded figures meet our requirements (or NAAS is able to fix the files to meet our requirements), the figure will be marked as "fixed" above. If NAAS is unable to fix the files, a red "failed" label will appear above. When NAAS has confirmed that the figure files meet our requirements, please download the file via the download option, and include these NAAS processed figure files when submitting your revised manuscript.

# Author comment: Author response to Decision Letter 1

**DOI:** 10.1371/journal.pmen.0000645.r003

13 Feb 2026

Attachment Submitted filename: 260213 Response to reviewers comments.docx

# Peer review: Decision Letter 1

**DOI:** 10.1371/journal.pmen.0000645.r004

**Authors:** Emma Campbell, Ilse Bloom

9 Apr 2026

PMEN-D-25-00458R1

The psychosocial impact of disability and stigma among persons affected by leprosy and lymphatic filariasis in India

PLOS Mental Health

Dear Dr. van Wijk,

Thank you for submitting your manuscript to PLOS Mental Health. After careful consideration, we feel that it has merit but does not fully meet PLOS Mental Health’s publication criteria as it currently stands. Therefore, we invite you to submit a revised version of the manuscript that addresses the points raised during the review process.

The manuscript has been further evaluated by three reviewers, and their comments are available below.

The reviewers have requested greater methodological detail and further interpretation of some of the findings.

Could you please carefully revise the manuscript to address all comments raised?

Please submit your revised manuscript by May 23 2026 11:59PM. If you will need more time than this to complete your revisions, please reply to this message or contact the journal office at mentalhealth@plos.org. When you're ready to submit your revision, log on to https://www.editorialmanager.com/pmen/ and select the 'Submissions Needing Revision' folder to locate your manuscript file.

Please include the following items when submitting your revised manuscript:

- A letter that responds to each point raised by the editor and reviewer(s). You should upload this letter as a separate file labeled 'Response to Reviewers'.
- A marked-up copy of your manuscript that highlights changes made to the original version. You should upload this as a separate file labeled 'Revised Manuscript with Track Changes'.
- An unmarked version of your revised paper without tracked changes. You should upload this as a separate file labeled 'Manuscript'.

Guidelines for resubmitting your figure files are available below the reviewer comments at the end of this letter.

As the corresponding author, your ORCID iD is verified in the submission system and will appear in the published article. PLOS supports the use of ORCID, and we encourage all coauthors to register for an ORCID iD and use it as well. Please encourage your coauthors to verify their ORCID iD within the submission system before final acceptance, as unverified ORCID iDs will not appear in the published article. *Only*  the individual author can complete the verification step; PLOS staff *cannot*  verify ORCID iDs on behalf of authors.

We look forward to receiving your revised manuscript.

Kind regards,

Ilse Bloom

Staff Editor

PLOS Mental Health

Journal Requirements:

If the reviewer comments include a recommendation to cite specific previously published works, please review and evaluate these publications to determine whether they are relevant and should be cited. There is no requirement to cite these works unless the editor has indicated otherwise.

Additional Editor Comments (if provided):

[Note: HTML markup is below. Please do not edit.]

Reviewers' comments:

Reviewer's Responses to Questions

**Comments to the Author**

1. If the authors have adequately addressed your comments raised in a previous round of review and you feel that this manuscript is now acceptable for publication, you may indicate that here to bypass the “Comments to the Author” section, enter your conflict of interest statement in the “Confidential to Editor” section, and submit your "Accept" recommendation.

Reviewer #4: All comments have been addressed

Reviewer #5: (No Response)

Reviewer #6: (No Response)

**********

2. Does this manuscript meet PLOS Mental Health’s publication criteria? Is the manuscript technically sound, and do the data support the conclusions? The manuscript must describe methodologically and ethically rigorous research with conclusions that are appropriately drawn based on the data presented.

Reviewer #4: Yes

Reviewer #5: Yes

Reviewer #6: Yes

**********

3. Has the statistical analysis been performed appropriately and rigorously?

Reviewer #4: Yes

Reviewer #5: Yes

Reviewer #6: Yes

**********

4. Have the authors made all data underlying the findings in their manuscript fully available (please refer to the Data Availability Statement at the start of the manuscript PDF file)?

The PLOS Data policy requires authors to make all data underlying the findings described in their manuscript fully available without restriction, with rare exception. The data should be provided as part of the manuscript or its supporting information, or deposited to a public repository. For example, in addition to summary statistics, the data points behind means, medians and variance measures should be available. If there are restrictions on publicly sharing data—e.g. participant privacy or use of data from a third party—those must be specified.

Reviewer #4: Yes

Reviewer #5: Yes

Reviewer #6: Yes

**********

5. Is the manuscript presented in an intelligible fashion and written in standard English?

PLOS Mental Health does not copyedit accepted manuscripts, so the language in submitted articles must be clear, correct, and unambiguous. Any typographical or grammatical errors should be corrected at revision, so please note any specific errors here.

Reviewer #4: Yes

Reviewer #5: Yes

Reviewer #6: Yes

**********

6. Review Comments to the Author

Please use the space provided to explain your answers to the questions above. You may also include additional comments for the author, including concerns about dual publication, research ethics, or publication ethics. (Please upload your review as an attachment if it exceeds 20,000 characters)

Reviewer #4: Thank you for the opportunity to review this important and well-conducted study. The manuscript addresses a highly relevant topic—psychosocial impact, stigma, and disability among persons affected by leprosy and lymphatic filariasis (LF)—and uses a relatively large sample with validated tools. The inclusion of both conditions and a community comparison group is a strength of this paper, and the findings have clear implications for integrated NTD and mental health programmes.

Here are some suggestions

The sampling combines purposive (district selection, community controls) and random approaches (cases). Community controls were recruited only in Bokaro, while cases came from both Bokaro and Jaunpur. This may introduce a selection bias and limit the comparison. There needs to be a line of justification.

A conceptual framework stating the relationships between stigma, depression, participation, and mental wellbeing will improve the interpretations.

Mention who administered the tools and what was the language used.

Explain the finding higher education associated with poorer mental wellbeing (leprosy group) - suggestions- increased awareness and/or unmet expectations. Explain leprosy shows higher stigma; LF shows different stigma domains.

recommendations could be more specific and actionable.

Minor spelling mistakes can be corrected. (participants, restriction)

Reviewer #5: Dear authors, congratulations on your work. The result of your work will enrich the existing evidence regarding stigma toward people with leprosy and lymphatic filariasis in India. I found no major revisions in this revision 1 document. However, I still have some input for the authors to consider, listed below.

1. Background

• Are there specific impacts, stigmas, or previous reports regarding disability in Bokaro and Jaunpur districts that justify selecting these locations? If so, please include them in the introduction or the methods section.

2. Methodology

• Why was the additional study for community members performed only in the Bokaro district?

• Given the total of 57 items across the four measuring instruments, what was the estimated data collection time per participant? Is it possible to mention in the manuscript?

Reviewer #6: Review comments and concerns.

This is a relevant study exploring the psychosocial impact of disability and stigma among persons affected by leprosy and lymphatic filariasis, and I acknowledge the authors on their original research work. I appreciate the chance to review this work and trust that my feedback will provide valuable insights.

General comments:

As I read the manuscript, I found it to be well written and informative. It contains a clear abstract, background, and methodology, with only minor comments and a good presentation of the results. The discussion nicely addresses the main findings. Additionally, the strengths and limitations of the study are appreciated, as is the conclusion. However, the conclusion could be improved by considering other recommendations to make it more comprehensive.

Author Summary

Line 64: It would be better to add 'psychological' to mean medical and social support. This is because your title mainly focuses on psychosocial issues.

Background

Line 68: According to the reference, there are 22 NTDs rather than 21. It would be better to correct this data.

Line 83: It is very important to describe that parasite. Therefore, it is valuable to add Wuchereria bancrofti, Brugia malayi, and Brugia timori.

Line 85: It would be better to say 'the parasite' instead of 'the diseases.'

Line 86: Make it clear that LF also affects the arm and breast, depending on the affected site.

Lines 87 and 88 need a reference.

Methodology

Study Design

Line 132: Study design. This includes the study setting and period, so include these in the subtitle.

Line 134: Does your study design indicate an analysis of psychosocial outcomes and the influence of disability and stigma? It would be better to consider the difference between correlation and causation here.

Line 137: You purposively selected two areas due to their high endemicity. Do you think the results can be generalized to low-endemic areas as well?

It would be better to change the location of lines 140–143 to the data collection section/subtitle, unless you have a concrete reason for including them here.

Study Population

Sampling

Line 149: What type of random sampling did you use for the selection? Additionally, how many people with disabilities were found in the two districts, and how was the collected sample allocated to each site? This needs further clarification.

Line 154: What impact does excluding people with serious mental health problems have on the validity and results?

Line 156: Only community members were selected by gender. Do you think age and educational level are irrelevant factors in the validity of the control? Why not consider these factors as well?

Line 159: Why were family members, relatives, and direct neighbors excluded?

Sample size

Line 164: Why were community members selected only in the Bokaro district? How does this align with the context-specific cut-off values for this measurement presented on line 142?

Data Collection

Line 200: Delete one of the 35 at the end of the sentence.

Results

In Table 1: Please delete the 'grade 0 disability' row value. To be consistent, change 'n/a' to 'N/A'.

Mental well-being, depression and participation restriction

Line 252. In what ways do the psychometric properties and score distributions of the Participation Scale (PSSS) differ from those of the PHQ-9? How does this influence the choice between mean and median as a measure of central tendency?

Line 255: Why are you using the median for the PSSS?

In Table 2: Why is it important to report the mean with CI and the median with IQR in the same table?

Stigma

Lines 259–260: Why is the median used for SSS?

Line 264: It is better to report by grade number than to report lower lymphoedema grades.

In Table 3, why is the mean per question reported for experienced stigma, disclosure concern, internalized stigma, and anticipated stigma, rather than the mean of all questions and the median of each subscale?

In addition, when reporting these in the table, it is preferable to describe the lower and upper ranges of the mean subscale scores in the Methods section.

Factors influencing psychosocial well-being

Line 277: It says, 'Associations between the psychosocial outcomes and stigma are visualized in Figure 1,' but, as described above, the figure is not included in the document.

Persons affected by Leprosy

From line 278: I understand that there is no association between stigma and mental well-being and that persons affected by leprosy are indirectly part of the description. If so, it would be better to describe this in the final part of the paragraph alongside its P-value.

Persons affected by lymphatic filariasis

However, in lines 290 and 291. I have seen the reputation of ideas for people affected by leprosy, stigma, and depression under the 'Persons Affected by Lymphatic Filariasis' sub-section. So, delete this part.

Line 293 or Table 4. It is good to present it in landscape format, and for the mean and median, present one row for each variable and one line for the confidence interval (CI) and interquartile range (IQR) accordingly. To avoid writing sociodemographic variables twice for persons affected by leprosy and LF, it is better to write them in front of the first column: sex, age, marital status, and so on. This will help readers to grasp the information easily, as they need a clear table presentation.

Again, for the Table 4 result, you have not described what type of statistical analysis you used.

Stigma and other determinants related to psychosocial well-being

Line 313: Table 5. Why is a multilevel model being run for a combined sample? How do you categorize visible and non-visible disabilities here? Also, why do you use the symbol β instead of B for beta?

Discussion

Line 358: Koschorke et al. is reference #42, not #41. Reference #41 is by Somar, Waltz, and van Brakel.

Conclusion

Lines 421 – 428. It is vital to address mental well-being, depression, restricted participation, and stigma by integrating the various intervention methods you listed. However, these are not the only interventions. As previously mentioned, psychosocial problems are related to disability and stigma. Therefore, raising awareness and implementing religious, psychosocial, economic, holistic, and family-based interventions can significantly alleviate such problems, as evidenced by research conducted in various areas. As a recommendation, it would be better to make the approach more comprehensive.

**********

7. PLOS authors have the option to publish the peer review history of their article (what does this mean?). If published, this will include your full peer review and any attached files.

**Do you want your identity to be public for this peer review?**  If you choose “no”, your identity will remain anonymous but your review may still be made public.

For information about this choice, including consent withdrawal, please see our Privacy Policy.

Reviewer #4: **Yes:**  Joydeepa Darlong

Reviewer #5: **Yes:**  Yoslien Sopamena

Reviewer #6: **Yes:**  Moges Wubie Aycheh

**********

[NOTE: If reviewer comments were submitted as an attachment file, they will be attached to this email and accessible via the submission site. Please log into your account, locate the manuscript record, and check for the action link "View Attachments". If this link does not appear, there are no attachment files.]

-->Figure Resubmissions:

While revising your submission, we strongly recommend that you use PLOS’s NAAS tool (https://ngplosjournals.pagemajik.ai/artanalysis) to test your figure files. NAAS can convert your figure files to the TIFF file type and meet basic requirements (such as print size, resolution), or provide you with a report on issues that do not meet our requirements and that NAAS cannot fix.

After uploading your figures to PLOS’s NAAS tool - https://ngplosjournals.pagemajik.ai/artanalysis, NAAS will process the files provided and display the results in the "Uploaded Files" section of the page as the processing is complete. If the uploaded figures meet our requirements (or NAAS is able to fix the files to meet our requirements), the figure will be marked as "fixed" above. If NAAS is unable to fix the files, a red "failed" label will appear above. When NAAS has confirmed that the figure files meet our requirements, please download the file via the download option, and include these NAAS processed figure files when submitting your revised manuscript.

# Author comment: Author response to Decision Letter 2

**DOI:** 10.1371/journal.pmen.0000645.r005

21 May 2026

Attachment Submitted filename: 260521 Response to reviewers comments.docx

# Editor report: Decision Letter 2

**DOI:** 10.1371/journal.pmen.0000645.r006

**Authors:** Emma Campbell, Ilse Bloom, Ibrahim Luberenga

8 Jun 2026

The psychosocial impact of disability and stigma among persons affected by leprosy and lymphatic filariasis in India

PMEN-D-25-00458R2

Dear Robin Van Wijk,

We are pleased to inform you that your manuscript 'The psychosocial impact of disability and stigma among persons affected by leprosy and lymphatic filariasis in India' has been provisionally accepted for publication in PLOS Mental Health.

Before your manuscript can be formally accepted you will need to complete some formatting changes, which you will receive in a follow up email. A member of our team will be in touch with a set of requests.

Please note that your manuscript will not be scheduled for publication until you have made the required changes, so a swift response is appreciated.

IMPORTANT: The editorial review process is now complete. PLOS will only permit corrections to spelling, formatting or significant scientific errors from this point onwards. Requests for major changes, or any which affect the scientific understanding of your work, will cause delays to the publication date of your manuscript.

If your institution or institutions have a press office, please notify them about your upcoming paper to help maximize its impact. If they'll be preparing press materials, please inform our press team as soon as possible -- no later than 48 hours after receiving the formal acceptance. Your manuscript will remain under strict press embargo until 2 pm Eastern Time on the date of publication. For more information, please contact mentalhealth@plos.org.

Thank you again for supporting Open Access publishing; we are looking forward to publishing your work in PLOS Mental Health.

Best regards,

Ibrahim Luberenga, M.D

Guest Editor

PLOS Mental Health

***********************************************************

Reviewer Comments (if any, and for reference):
